# How Vaccinating People Living With HIV May Guide bNAb‑Based Vaccines

**DOI:** 10.1002/jia2.70119

**Published:** 2026-05-15

**Authors:** Penny L. Moore, Leonidas Stamatatos, Alexandra Trkola

**Affiliations:** ^1^ Antibody Immunity Research Unit, an Extramural Unit of the South African Medical Research Council Faculty of Health Sciences, University of the Witwatersrand Johannesburg South Africa; ^2^ National Institute for Communicable Diseases of the National Health Laboratory Services Johannesburg South Africa; ^3^ Wits Infectious Diseases and Oncology Research Institute, Faculty of Health Sciences University of the Witwatersrand Johannesburg South Africa; ^4^ Centre for the AIDS Programme of Research in South Africa Durban South Africa; ^5^ Fred Hutchinson Cancer Center Vaccine and Infectious Disease Division Seattle Washington USA; ^6^ Department of Global Health University of Washington Seattle Washington USA; ^7^ Institute of Medical Virology University of Zurich Zurich Switzerland

1

The HIV pandemic continues to pose a major global public health challenge. Despite the success of antiretroviral therapy (ART), access to lifelong treatment for all people living with HIV (PLWH) worldwide is not universal or affordable, and is compromised by recent changes in the funding landscape, highlighting the need for preventative vaccines. Two large efficacy trials of a single broadly neutralizing antibody (bNAb), VRC01, provided evidence that bNAbs can block acquisition by antibody‐sensitive HIV strains, corroborating previous studies in non‐human primates. These trials provided proof‐of‐principle for antibody‐mediated protection, and generated essential biological insights that strengthened HIV vaccine development [1].

Recent years have seen extraordinary advances in HIV immunogen design. Early phase trials of “germline‑targeting” immunogens (aimed at activating the extremely rare precursors of bNAbs) in HIV‐naive participants have shown engagement of these naive B‑cell receptors, and early maturation towards their broadly neutralizing forms [2, 3, 4]. However, the vaccination schema needed to go from this early stage of B‐cell activation to complete bnAb maturation remains to be established, and will require extensive Phase I testing and validation in Phase II/III efficacy trials. Trials of bNAb‐inducing vaccines have historically been tested in HIV‐naive participants, the target population for a preventative vaccine. However, given that providing state‐of‐the‐art prevention, including ART, to HIV‐naive individuals participating in vaccine trials is an undeniable ethical necessity, the logistical challenges of conducting vaccine trials in populations with low rates of HIV transmission are immense. In this era of highly effective ART, only extremely large‐scale trials have the statistical power to establish vaccine efficacy [5]. This provides an additional hurdle to vaccine development.

In this Viewpoint, we argue that vaccine trials focused on bNAb induction should expand to include PLWH on ART to advance both preventative and therapeutic vaccines [6, 7] (Figure 1A). Assessing bNAb‐inducing vaccines in PLWH may address several vaccine design challenges for bNAb maturation. While germline‐targeting HIV‐1 envelope (Env)‐derived immunogens can activate naive B cells expressing the unmutated (germline) forms of bnAbs, these responses are low titre, and the resulting antibodies are only partially mutated with limited neutralizing activity. Their full maturation will require boosting with a series of tailored, heterologous immunogens. While pre‐clinical studies in mice engineered to express human B cell receptors will inform the design of these booster immunogens [8], iterative testing of multiple constructs and regimens in HIV‑naive volunteers will be required, a slow, expensive process that is fundamentally rate‐limiting.

**FIGURE 1:**
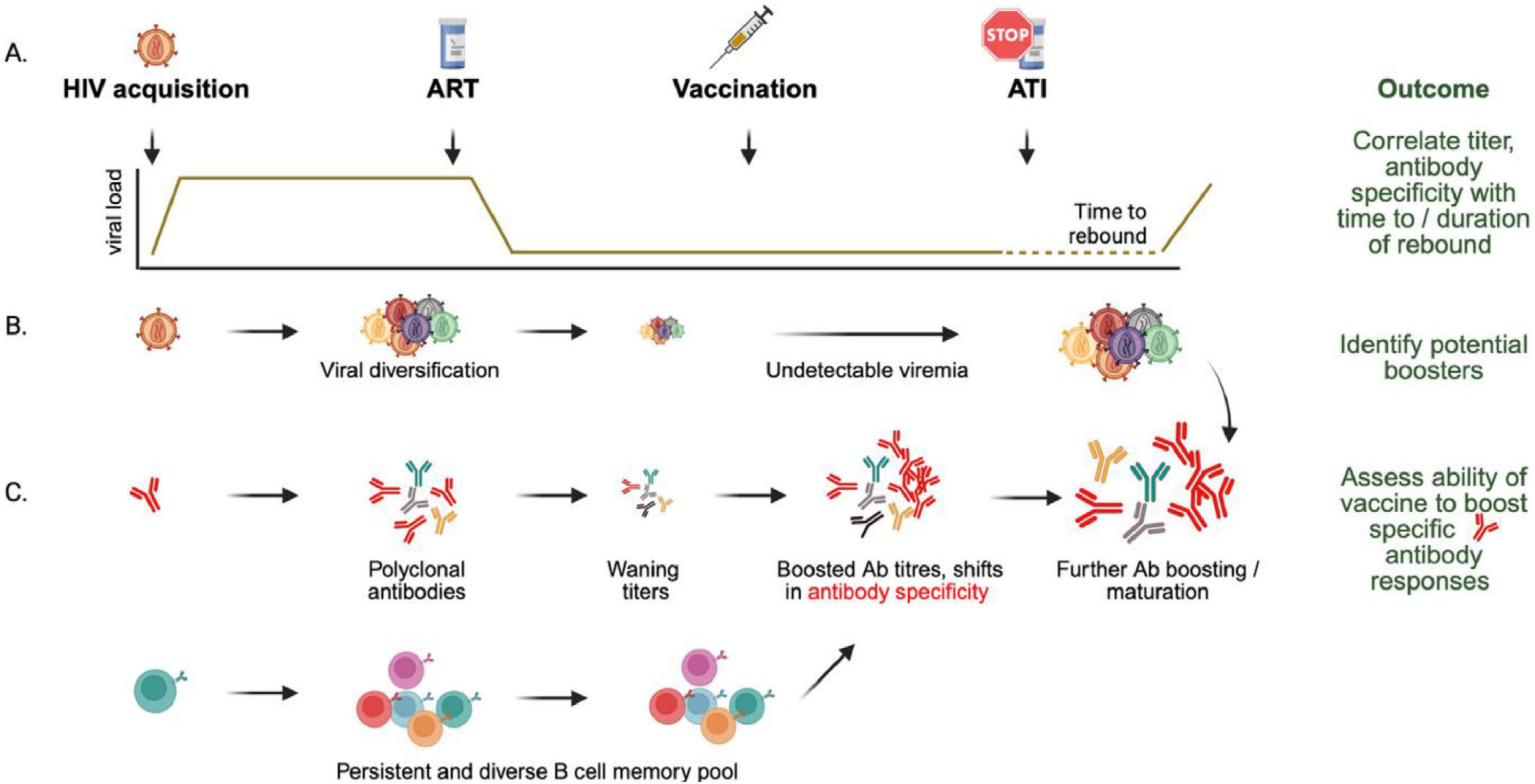
Schematic showing key events and outcomes of the vaccination of people living with HIV (PLWH) followed by analytic treatment interruption (ATI). (A) Viral load changes from HIV acquisition through to antiretroviral therapy (ART) initiation, vaccination and ATI. The dotted line indicates the variable period from stopping ART to viral rebound. (B) Following acquisition, viral diversification results in a heterogeneous swarm of viral variants, which seed the reservoir upon ART initiation but rebound following an ATI. (C) Schematic showing antibody kinetics and titres after HIV acquisition. Initial strain‐specific antibody responses evolve to form a polyclonal response, creating a persistent and diverse B‐cell memory pool. Upon ART initiation, antibody titres will wane, though memory B cells will persist. Following vaccination, antibody titres will be boosted again, and vaccine‐targeted specificities may be enriched, resulting in shifts in specificity. An ATI will likely drive further increases in antibody titres and maturation towards bNAbs, and deep sequencing of rebounding viral variants may enable the identification of potential boosters.

During HIV infection, there is significant viral diversification (Figure 1B). The HIV‐1 Env trimer is also conformationally labile, sampling a continuum of closed and open states, generating a spectrum of Env conformations on virions. Lastly, immunological decoys such as shed gp120 and gp41 stumps expand the range of envelope forms to which the immune system of PLWH is exposed. Therefore, any viraemic period results in immunologically “primed” individuals with diverse Env‐specific memory B‐cell responses that persist for years, even if subsequent ART suppresses viraemia (Figure 1C) [3].

Single‑ or short‑course vaccination of PLWH on ART provides an opportunity to probe this highly primed landscape. Vaccinating PLWH may reveal whether a candidate Env immunogen stimulates de novo bNAb precursor responses, restimulates bNAb‑like lineages or drives other immunodominant responses [6] (Figure 1C). For vaccines designed to trigger specific bNAbs, such as CD4 binding site bNAbs, sequencing of the B cell repertoire before and after vaccination will confirm whether such antibodies are enriched (Figure 1B). Furthermore, these trials can explore how prior infection history, cumulative viral load, or the presence of bNAbs targeting the same or other epitopes as the vaccine, influences vaccine responsiveness [6]. Comparing elite neutralizers with non‐bNAb donors may contribute to understanding intrinsic barriers to neutralizing breadth, which only occurs after years of untreated acquisition in some PLWH. Comparison of PLWH treated early versus later after acquisition may provide insights into the extent of priming required. These basic immunological insights will inform vaccine design strategies.

Performing these studies in different geographies and demographies is critical. These trials may begin to link vaccine outcomes to viral clade, duration of infection and host factors [6]. Viral clade impacts bNAb induction. For example, clade B HIV‐1 triggers more CD4 binding site responses, whereas V2‐apex responses are more common in clade C infection [9]. Host immunogenetics also impact antibody responses to several pathogens, including HIV‐1. Germline‐targeting HIV trials for VRC01‐class bNAbs rely on VH1‐2*02 and *04 immunoglobulin alleles, and people lacking them cannot respond to these vaccines [2]. However, global immunogenetic databases are biased towards those of white, European ancestry and may miss 70%–90% of immunoglobulin gene diversity, and alternative pathways to generating CD4 binding site bNAbs may exist [10, 11, 12]. Together, these “knowns” and “unknowns” suggest that including individuals with diverse host backgrounds, and living with different viral clades, may reveal novel immunological pathways to bNAbs [6]. Crucially, many of these insights could be obtained after a single immunization, providing a high‑information, low‑burden alternative to multi‑dose, multi‑year programmes in HIV‑naive populations.

A second component of vaccinating PLWH includes analytic treatment interruptions (ATIs) after vaccination. The heterogeneous viral quasispecies that exist in PLWH are numerically more likely than any single immunogen to contain an Env variant that binds bnAb precursors elicited by a germline‐targeting immunogen. A carefully monitored ATI allows viral rebound, during which emerging Envs will interact with partially matured B‑cell lineages elicited by the vaccine. By sequencing Env from rebounding virus, alongside studies of B‑cell receptor repertoires and functional antibody responses, it may be possible to identify Envs that function as in vivo “boosters” for vaccine‑elicited precursors. These Envs could be employed as booster immunogens in HIV‑naive individuals [7].

ATIs may also inform correlates of protection, thus far estimated from passive immunization prevention settings. The HIV field lacks robust benchmarks for vaccine‑induced control of established HIV acquisition or viral rebound after ART interruption [1, 13, 14]. Experimental medicine trials in HIV‐naive populations provide deep immunological insights but are limited to small numbers of participants. Relating neutralization titres, epitope specificities and B‑cell lineage features to the timing and magnitude of rebound may define protective antibody profiles. Vaccine‑plus‑ATI studies in PLWH could extend correlates of protection beyond acquisition to early/prolonged control of rebound viraemia.

There are ethical considerations as well: vaccination while on ART needs to be clearly communicated as experimental and not an alternative to ART. ATI‑based studies must carefully follow treatment‑interruption protocols, with appropriate eligibility criteria, close monitoring and predefined ART restart thresholds [15].

Evaluation of novel vaccine concepts in PWLH on ART should be developed in parallel with, and not in place of, trials in HIV‑naive individuals. However, the field would be remiss in not incorporating such studies within the global HIV vaccine strategy, as it will undoubtedly accelerate the development of both preventive and therapeutic vaccine strategies.

## Author Contributions

**Penny L. Moore**: Conceptualization, writing – original draft, visualization, writing – review and editing, methodology. **Leonidas Stamatatos**: Conceptualization, writing – original draft, writing – review and editing, visualization, methodology. **Alexandra Trkola**: Conceptualization, writing – original draft, writing – review and editing, visualization, methodology.

## Funding

PLM is supported by the South African Research Chairs Initiative of the Department of Science and Innovation and National Research Foundation of South Africa, the South African Medical Research Council SHIP program, and the Centre for the AIDS Program of Research (CAPRISA) and received long‐term funding from USAID. PLM and AT are supported by the Gates Foundation. LS is funded by the US National Institutes for Health and the Gates Foundation.

## Conflicts of Interest

PLM, LS and AT are conducting trials in PLWH, with funding from the Gates Foundation and the National Institutes for Health.
